# Effect of body mass index on survival after spinal cord injury

**DOI:** 10.3389/fneur.2023.1269030

**Published:** 2024-01-26

**Authors:** Nader Fallah, Vanessa K. Noonan, Nancy P. Thorogood, Brian K. Kwon, Marcel A. Kopp, Jan M. Schwab

**Affiliations:** ^1^Praxis Spinal Cord Institute, Blusson Spinal Cord Centre, Vancouver, BC, Canada; ^2^Department of Medicine, University of British Columbia, Vancouver, BC, Canada; ^3^Department of Orthopaedics, Vancouver Spine Surgery Institute, University of British Columbia, Vancouver, BC, Canada; ^4^International Collaboration on Repair Discoveries (ICORD), University of British Columbia, Vancouver, BC, Canada; ^5^Department of Neurology and Experimental Neurology, Clinical and Experimental Spinal Cord Injury Research, Charité – Universitätsmedizin Berlin, Berlin, Germany; ^6^QUEST-Center for Transforming Biomedical Research, Berlin Institute of Health, Berlin, Germany; ^7^Department of Neurology, Spinal Cord Injury Division, The Ohio State University, Wexner Medical Center, Columbus, OH, United States; ^8^Belford Center for Spinal Cord Injury, Departments of Physical Medicine and Rehabilitation and Neuroscience, The Ohio State University, Wexner Medical Center, Columbus, OH, United States

**Keywords:** acute spinal cord injury, body mass index, mortality risk, Charlson comorbidity index, injury severity score

## Abstract

**Introduction:**

Increased mortality after acute and chronic spinal cord injury (SCI) remains a challenge and mandates a better understanding of the factors contributing to survival in these patients. This study investigated whether body mass index (BMI) measured after acute traumatic SCI is associated with a change in mortality.

**Methods:**

A prospective longitudinal cohort study was conducted with 742 patients who were admitted to the Acute Spine Unit of the Vancouver General Hospital between 2004 and 2016 with a traumatic SCI. An investigation of the association between BMI on admission and long-term mortality was conducted using classification and regression tree (CART) and generalized additive models (spline curves) from acute care up to 7.7 years after SCI (chronic phase). Multivariable models were adjusted for (i) demographic factors (e.g., age, sex, and Charlson Comorbidity Index) and (ii) injury characteristics (e.g., neurological level and severity and Injury Severity Score).

**Results:**

After the exclusion of incomplete datasets (*n* = 602), 643 patients were analyzed, of whom 102 (18.5%) died during a period up to 7.7 years after SCI. CART identified three distinct mortality risk groups: (i) BMI: > 30.5 kg/m^2^, (ii) 17.5–30.5 kg/m^2^, and (iii) < 17.5 kg/m^2^. Mortality was lowest in the high BMI group (BMI > 30.5 kg/m^2^), followed by the middle-weight group (17.5–30.5 kg/m^2^), and was highest in the underweight group (BMI < 17.5 kg/m^2^). High BMI had a mild protective effect against mortality after SCI (hazard ratio 0.28, 95% CI: 0.09–0.88, *p* = 0.029), concordant with a modest “obesity paradox”. Moreover, being underweight at admission was a significant risk factor for mortality up to 7.7 years after SCI (hazard ratio 5.5, 95% CI: 2.34–13.17, *p* < 0.001).

**Discussion:**

Mortality risk (1 month to 7.7 years after SCI) was associated with differences in BMI at admission. Further research is needed to better understand the underlying mechanisms. Given an established association of BMI with metabolic determinants, these results may suggest unknown neuro-metabolic pathways that are crucial for patient survival.

## Introduction

Mortality after spinal cord injury (SCI) remains a substantial challenge (1). While infections and septic conversions remain the main causes, so far unknown reasons may drive mortality risk. Obesity is a well-characterized modifiable risk factor for vascular disease, which warrants control for primary and secondary prevention of stroke (2, 3) and represents a substantial challenge. Obesity and cardiometabolic risk markers are frequently pre-existent in patients with acute SCI (4).

In contrast to the deleterious chronic effects of obesity, ischemic brain injury studies have demonstrated that obese patients may have a lower acute mortality rate compared to their underweight counterparts (5, 6). This has been confirmed by *post-hoc* analysis of large trials (7), including the randomized, multicenter Field Administration of Stroke Therapy–Magnesium Study (8). The paradoxical phenomenon of lower mortality despite a higher risk of recurrent vascular insults in patients with obesity is referred to as the “obesity paradox” (9, 10). One explanatory reason is a catabolic state early after CNS injury being aggravated further by additional energy resources that are required for mounting a stress response and temperature rise in case of prevalent fever. The impaired ability to respond to these challenges due to a dysregulated, decentralized autonomic nervous system suggests the presence of non-homeostatic compensation strategies. In addition to cancer (11) and stroke, an obesity paradox has also been described in amyotrophic lateral sclerosis, where a lowered risk for disease progression or death has been observed in individuals with a high body mass index (BMI) (12, 13).

Acute SCI represents a life-threatening event triggering a profound stress response mirrored by hypercortisolism (14–16). Hypercortisolism indicates a stress response capable of mobilizing the body’s energy, which can decrease lean body and muscle mass. Applying a bed-to-benchside approach to understand causality, a recent study verified lesion-level-dependent hypercortisolism as a catabolic and systemic driver of muscle wasting/sarcopenia, contributing to early weight loss after SCI affecting the entire body, including non-denervated muscles above the lesion site (17). It appears that the acute time window after SCI is different from the chronic SCI phase. During the first 6–10 weeks post-injury, early weight loss and body fat reduction have been reported (18), verifying a prevailing catabolic state. Recent studies have determined the association of body mass with mortality occurring after this first catabolic phase. This includes data from the US-National SCI Model System Database examining mortality from 3 months to 1 year after SCI (19). A putative obesity-related protective effect, however, would be expected during the first 3 months after SCI, with concurrent weight loss and body fat reduction (18). In addition to this putative “protective” effect of high BMI, an entirely different pathophysiological response may be in effect in cases of low BMI or being “underweight,” which may also impact mortality.

To provide an integrative and comprehensive assessment of the role of nutritional status/body mass index ‘on admission’ after a traumatic SCI, we analyzed mortality over time from acute to chronic phases of SCI. Specifically, to test the hypothesis regarding the influence of BMI on mortality at different time points following SCI, we examined the association between admission BMI and mortality data at 1 month, 3 months, 1 year, and a long-term endpoint extending up to 7.7 years after SCI to analyze the dynamic association of admission BMI with mortality.

## Materials and methods

### Study and ethical approval

The study was approved by both the Vancouver Coastal Health Research Institute and the University of British Columbia Clinical Research Ethics Board. Data were collected from interviews and medical chart abstraction for individuals who consented to participate in the Rick Hansen Spinal Cord Injury Registry (expanded dataset) (20). In addition, data were collected via additional medical chart abstraction for individuals enrolled in RHSCIR under a consent waiver (minimal dataset).

### Study population, design, setting, and data variables

**Figure 1:**
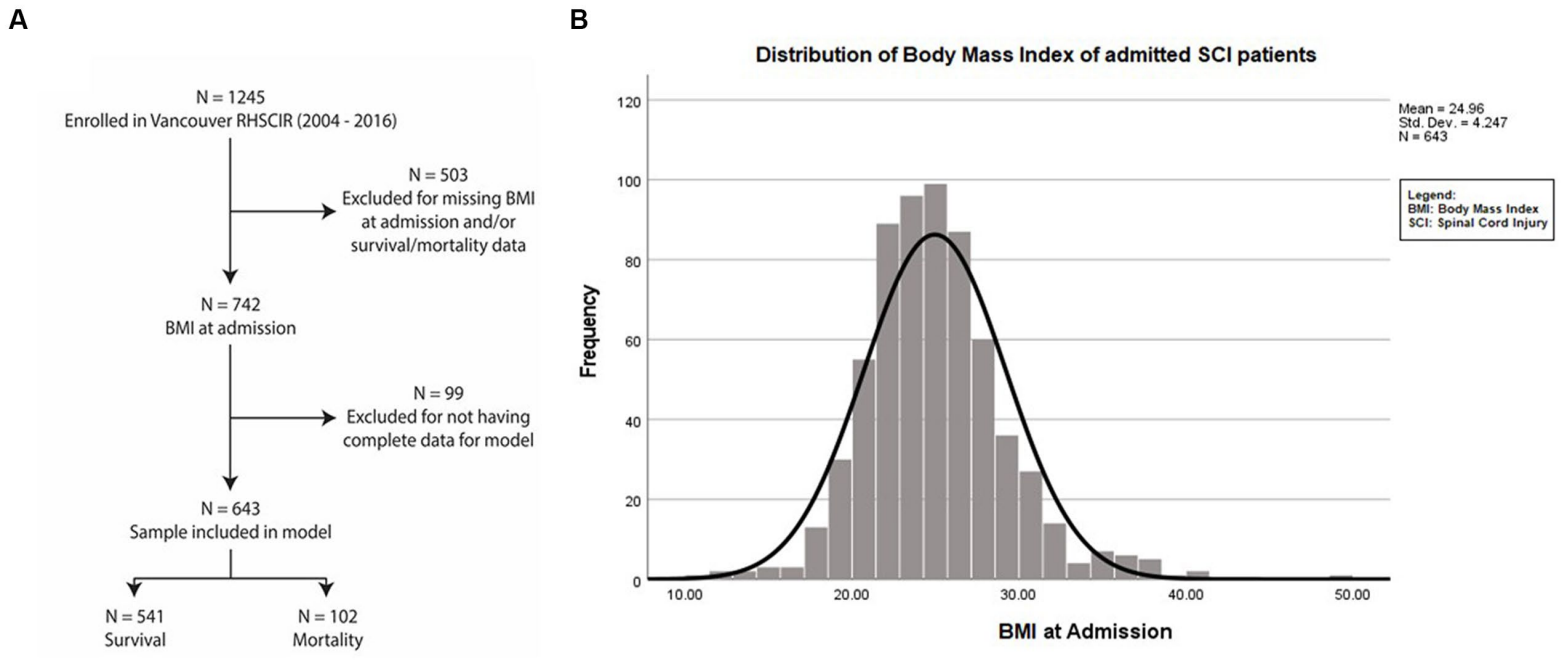
Dataset selection and enrolment. **(A)** Flowchart of patients admitted with traumatic SCI from 2004 to 2016 at the Acute Spine Unit. Among the 1,245 SCI participant datasets, 602 were excluded due to being incomplete. Of the remaining 643 participants, 102 died and 541 individuals survived up to 7.7 years after SCI. **(B)** Mean BMI at admission of the entire study population was 24.92 kg/m^2^ + 4.25 (SD).

This is a prospective longitudinal cohort study consisting of 1,245 acute SCI patients admitted to the Acute Spine Unit of the Vancouver General Hospital between 2004 and 2016 who were enrolled in the Rick Hansen Spinal Cord Injury Registry (RHSCIR) (20). Individuals with missing weight and/or height data at admission and mortality data were not included in this study (*n* = 503). Cases without complete data for model adjustment were excluded (*n* = 99) from the analysis. The sample used for the analysis was 643 (Figure 1A).

Age, sex, American Spinal Injury Association Impairment Scale (AIS), neurological level, Charlson Comorbidity Index (CCI), Injury Severity Score (ISS), body weight, and height were collected at admission to the Acute Spine Unit (21, 22). Pre-injury/admission body weight and height data were gathered by questioning the patients or their relatives. The BMI was calculated by dividing a person’s weight in kilograms by the square of a person’s height in meters (BMI = weight_kg_/height^2^_m_). The conventional BMI categories are underweight BMI < 18.5 kg/m^2^; normal weight BMI 18.5–24.9 kg/m^2^; overweight BMI 25.0–29.9 kg/m^2^; and obese, BMI ≥30.0 kg/m^2^ (2, 3). The outcome (mortality) was collected up to 7.7 years post-injury.

### Statistical modeling

Demographic and injury data were compared for the outcome (survival vs. mortality). Continuous variables including age, BMI, ISS, and CCI were analyzed using t-tests, and categorical variables such as sex, AIS, and neurological level groupings (i.e., Cl 1 to T1 vs. T2 to S5) were analyzed using chi-square tests. In a missing data analysis, demographic and injury characteristics were compared between groups that were defined based on the availability of BMI data.

To determine if BMI at admission was associated with mortality from acute care up to 7 years after SCI, predictive models were created using classification and regression tree (CART) analysis. This approach was chosen because the World Health Organization (WHO) obesity criteria (3) may be non-informative in patients with SCI (23, 24). CART accounted for the binomial distributions in the response variable and identified ‘nodes’, or subgroups that were most homogeneous with regards to the probability of mortality. These BMI subgroups were then applied to Kaplan–Meier curves and Cox regression. For comparability with other studies, we used BMI categories based on the WHO obesity criteria. In addition, Cox regression was conducted, categorizing BMI by the 10th, 11th–89th, and 90th percentiles in a sensitivity analysis. To determine the survival for each BMI category relative to the WHO normal or medium range for BMI, unadjusted and adjusted Kaplan–Meier curves and log-rank tests were calculated. All variables and stratified BMI categories were tested for proportionality assumptions (Schoenfeld residuals) before applying them to the Cox regression data in order to calculate mortality hazard ratios (MHRs). In this analysis, there was no evidence of time-varying effects. The model was adjusted for age, sex, AIS, neurological level (C1 to T1 vs. T2 to S5), and ISS (25–27). In total, two models were calculated [(i) using all variables and (ii) applying BMI only] for the analyses using the WHO BMI categories and the CART BMI categories.

To further elucidate the association between BMI and survival, we applied the generalized additive model (GAM) with cubic splines using the BMI continuous data instead of the BMI categories in a sensitivity analysis. Assessments were made over time at 1 month, 3 months, 1 year, and the long-term endpoint (7.7 years). A value of *p* of <0.05 was considered statistically significant, along with the 95% CI. All statistical analyses were performed using SPSS (version 26) and R × 64 (version 3.1).

## Results

We investigated mortality in patients admitted to the Acute Spine Unit of the Vancouver General Hospital (Figure 1A) with traumatic spinal cord injuries. An analysis comparing the cohort that was included vs. those excluded due to missing data revealed no relevant differences in sex or age. The included patients comprised slightly fewer cervical injuries and more individuals with AIS A injuries. Both groups had similar mortality rates (Supplementary Table S1).

The mean ± SD value of BMI for the patients during their acute admission was 24.96 ± 4.25 kg/m^2^ (Figure 1B). The distribution of (i) demographic factors (age, sex) and (ii) SCI characteristics [injury severity (AIS), neurological level (C1 to T1 vs. T2 to T12 vs. L1 to S5)], accompanying severity of ISS, and premorbid comorbidities using the CCI is described in Table 1.

**Table 1:** Demographic and injury data for the survival and mortality groups.

	Survival *n* = 541	Mortality *n* = 102	*p*-value
Age, mean ± SD	42.5 ± 17.9	63.0 ± 17.9	< 0.001
Sex, % male (*n*)	72.5% (407)	83.3% (85)	0.047
BMI kg/m^2^ at admission, mean ± SD	25.1 ± 4.2	24.2 ± 4.3	0.055
AIS at admission, % (*n*)			0.412
A	46% (251)	53% (54)	
B	13% (68)	15% (15)	
C	21% (112)	18% (18)	
D	20% (110)	15% (15)	
Neurological level of injury, % (*n*)			< 0.001
C1-T1	59% (318)	89% (91)	
T2-T12	28.5% (154)	8.8% (9)	
L1-S5	12.8% (69)	2% (2)	
CCI, mean ± SD	0.35 ± 0.83	0.83 ± 1.14	< 0.001
ISS, mean ± SD	27.1 ± 11.5	30.7 ± 16.9	0.044

Of the 643 patients, 102 (15.9%) died, and 541 survived up to 7.7 years post-SCI. The two groups were statistically different with regard to age, sex, neurological level, Charlson Comorbidity Index (CCI), and Injury Severity Score (ISS). The mortality group had a higher age, a higher number of men, a higher number of cervical injuries, and a higher CCI and ISS. A predictive model was used to adjust for: (i) demographic factors (age and sex) and (ii) SCI characteristics [level and completeness of injury (AIS; ASIA Impairment Scale)] as well as for comorbidities (CCI) and severity of polytrauma (ISS) associated with SCI. AIS, American Spinal Injury Association Impairment Scale; CCI, Charlson Comorbidity Index; ISS, Injury Severity Score; BMI, body mass index.

There were 446 individuals (69.3%) who were admitted directly to the center, while 197 (30%) were admitted indirectly. The time to admission was less than 24 h for 366 individuals (82%) in the direct admission group and 175 individuals (88.8%) in the indirect group. Rates of surgery were similar (88.8 and 91.4%) for the direct and indirect admitted patients, respectively.

During the follow-up period, 102 patients (15.8%) were deceased as of 2016, and the mean time to death post-injury was 25.85 ± 26.04 months. Mortality rates were 2.2% at 1 month, 5% at 6 months, 6.5% at 1 year, 12.1% at 3 years, and 15.9% at 7.7 years following SCI. The mortality group was characterized by being older, comprising more men, having more injuries to the cervical cord, and having a higher CCI and ISS.

### CART identified three distinct mortality-hazard (“risk”) groups

Comparing the survival of the 10th with the 90th BMI percentile of the study population, the mortality rate was higher. The survival time was shorter in the 10th percentile, where 15 of 64 patients died (24.4%) at a mean time of 81.4 (95% CI: 72.8–90.0) months after injury. In contrast, in the 90th percentile, 6 deaths occurred in 65 patients (9.2%) after 94.5 (95% CI: 89.9–99.1) months. In the 11th–89th percentile, 81 of 514 patients (15.8%) died at 88.3 (95% CI: 85.8–90.8) months after injury.

**Figure 2:**
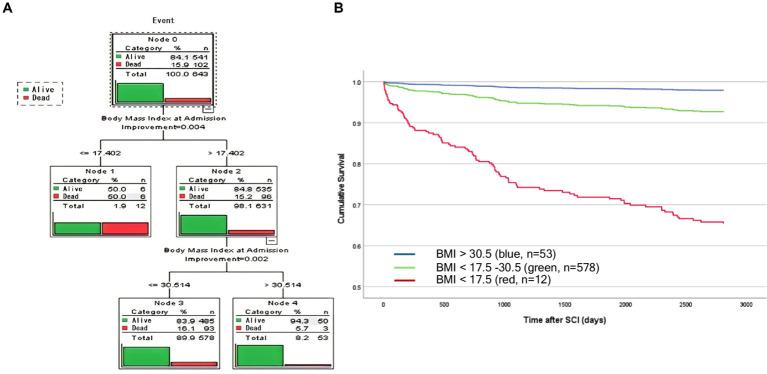
Analysis identified three distinct BMI groups using unbiased recursive partitioning (CART BMI categories). **(A)** A predictive model was developed by applying a classification and regression tree (CART) analysis. Survival was analyzed as an event following a binominal distribution [1 = mortality/event (red), vs. 0 = survival (green)]. Decision trees identified three cohorts that were most homogenous with regard to the probability of mortality. Survival was significantly lower in patients with a BMI < 17.5 kg/m^2^ (red) compared with patients with a BMI = 17.5 kg/m^2^ (Node 1 and 2). In the BMI cohort >17.5 kg/m^2^, patients with a BMI > 30.5 kg/m^2^ demonstrated lower mortality compared with patients with a BMI > 17.5–30.5 kg/m^2^ (Nodes 2 and 3). The overall number and percentage of deaths per group are listed and graphically illustrated in red vs. green (survival). **(B)** Linearized cumulative survival over time illustrated a protective effect of a higher BMI in a class (dose) dependent manner which occurs early and is long-lasting. Whereas, elevated mortality was observed in patients who were severely underweight (<17.5 kg/m^2^, red, *n* = 12), patients with a BMI of 17.5–30.5 kg/m^2^ (green, *n* = 578) or >30.5 kg/m^2^ (blue, *n* = 53) were protected mirrored by a less negative slope that nearly plateaus after 3 years.

To classify BMI categories based on the survival/mortality outcome, we applied CART to identify cohorts with different survival rates according to BMI. CART analysis identified three distinct subgroups (Figure 2A). Individuals with a BMI > 30.5 kg/m^2^ (blue, *n* = 53) demonstrated the lowest mortality, followed by patients with a BMI of 17.5–30.5 kg/m^2^ (green, *n* = 578), and the highest mortality was in patients with a BMI < 17.5 kg/m^2^ (red, *n* = 12) (Figure 2B). Survival analysis over time illustrated that the protective effects of higher BMI against mortality: (i) occurred in a dose-dependent manner, (ii) started early, and (iii) were long-lasting (Figure 2B). BMI groups are illustrated as Kaplan–Meyer curves after the Cox regression in Figure 2B. Comparison with WHO BMI categories confirmed the dose-dependent effect of BMI at admission on mortality (Supplementary Figure S2).

### The two effects on mortality: obesity and underweight

For the most accurate interpretation of the association between BMI and mortality (mortality hazard ratios) and to distinguish obesity/overweight from underweight-associated effects, Cox regression models were conducted, examining: (i) BMI bins as identified by recursive partitioning (Table 2) and (ii) BMI groups defined according to the WHO obesity criteria (Supplementary Table S2). Next, we examined whether the hazard ratios were different compared to the middle CART group (BMI 17.5–30.5 kg/m^2^, *n* = 578) or the WHO normal weight definition group (BMI 18.5–24.9 kg/m^2^, *n* = 325), respectively. The middle CART group (BMI 17.5–30.5 kg/m^2^, *n* = 578) and the WHO normal weight group (BMI 18.5–24.9 kg/m^2^, *n* = 325) were defined as the reference categories.

**Table 2:** Differential mortality risk for BMI groups identified by unbiased recursive partitioning (CART BMI groups).

Model	Variables	Hazard ratio(95% CI)		*p*-value
Univariable	BMI kg/m^2^ 17.5–30.5 (ref)			
	BMI kg/m^2^ < 17.5	4.18(1.83–9.57)		< 0.001
	BMI kg/m^2^ > 30.5	0.33(0.10–1.03)		0.056
Multivariable	Age (per 1 year increase)	1.07(1.06–1.08)		< 0.001
	Sex (male)	3.12(1.82–5.36)		< 0.001
	Neurological level (C1 to T1)	4.84(2.52–9.28)		< 0.001
	AIS A	3.496(1.83–6.68)		< 0.001
	AIS B	2.30(1.11–4.77)		0.024
	AIS C	1.68(0.84–3.35)		0.145
	AIS D	Ref		
	ISS (per 1 unit increase)	1.02(1.01–1.03)		0.003
	BMI 17.5–30.5 kg/m^2^ (ref)	Ref		
	BMI <17.5 kg/m^2^	5.55(2.34–13.17)		< 0.001
BMI >30.5 kg/m^2^	0.28(0.09–0.88)		0.029

Three groups of different body composition (BMI) were associated with a distinct mortality risk: (1) BMI 17.5–30.5 kg/m^2^ (reference), (2) BMI < 17.5 kg/m^2^, (3) BMI > 30.5 kg/m^2^. BMI group 2 had a 5.5-fold mortality risk compared to BMI group 1. By contrast, BMI group 3 had a 28% lower mortality risk compared to BMI group 1. AIS, American Spinal Injury Association Impairment Scale; ISS, Injury Severity Score; BMI, Body Mass Index. CART Categories: BMI < 17.5 (*N* = 12); BMI 17.5–30.5 (*N* = 578); BMI > 30.5 (*N* = 53).

Compared to the reference category (middle weight), the high-range BMI group identified by CART (BMI > 30.5 kg/m^2^, *n* = 53) displayed a significant decrease in mortality risk by 28% (HR 0.28, 95% CI 0.09–0.88, *p* = 0.029). In the obese WHO group (BMI ≥ 30.0 kg/m^2^, *n* = 67), the mortality risk was also significantly reduced to 32% (HR 0.32, 95% CI 0.14–0.76, *p* = 0.009) compared to the normal weight WHO group. In the overweight WHO group (BMI 25.0–29.9 kg/m^2^, *n* = 227), the effect on mortality was much weaker (HR 0.65, 95% CI 0.42–1.01, *p* = 0.053) compared to the medium/normal range BMI group.

By contrast, the low-range BMI group identified by the CART (BMI < 17.5 kg/m^2^, *n* = 12) likewise demonstrated a significantly elevated risk of mortality (HR 5.55, 95% CI 2.34–13.17, *p* < 0.001) compared to the CART-based mid-range BMI group (BMI 17.5–30.5 kg/m^2^). The underweight group, defined by the WHO criteria (BMI < 18.5 kg/m^2^, *n* = 24) was characterized by a significantly increased risk of mortality (HR 2.43, 95% CI 1.17–5.03, *p* = 0.017) compared to the WHO medium/normal weight group.

The three CART-defined BMI groups revealed a differing distribution based on the neurological impairment on admission (baseline), where there were more incomplete patients with SCI in the low-range BMI group and more cases with cervical SCI present in the middle BMI group. Other baseline characteristics that were slightly different between the groups were the CCI and ISS (Table 3). However, the differences in the neurological level distribution across the BMI groups had no effect on BMI-associated mortality (Supplementary Table S3).

**Table 3:** Baseline characteristics stratified for the CART BMI groups.

	BMI < 17.5 kg/m^2^ *n* = 12	BMI 17.5–30.5 kg/m^2^ *n* = 578	BMI > 30.5 kg/m^2^ *n* = 53
Age (years), mean ± SD	47.25 ± 21.97	45.58 ± 19.68	47.11 ± 15.38
Sex, % male (*n*)	75% (9)	76.5% (442)	77.4% (41)
BMI kg/m^2^at admission, mean ± SD	14.47 ± 2.21	24.34 ± 2.91	34.1 ± 3.59
AIS at admission, % (*n*)			
A	33.3% (4)	47.8% (276)	47.2% (25)
B	25% (3)	11.4% (66)	26.4% (14)
C	25% (3)	20.4% (118)	17% (9)
D	16.7% (2)	20.4% (118)	9.4% (5)
Cervical injury (C1 to T1), % (*n*)	50% (6)	64.9% (375)	52.8% (28)
CCI, mean ± SD	0.67 ± 1.23	0.41 ± 0.86	0.60 ± 1.17
ISS, mean ± SD	31.67 ± 17.7	27.67 ± 12.61	27.13 ± 10.72

Three body composition (BMI) groups were calculated based on CART: (1) BMI < 17.5 kg/m^2^; (2) BMI 17.5–30.5 kg/m^2^, and (3) BMI > 30.5 kg/m^2^. The three groups differed by ASIA Impairment Scale (AIS), neurological level (C1-T1), Charlson Comorbidity Index (CCI), and Injury Severity Score (ISS). AIS, American Spinal Injury Association Impairment Scale; BMI, body mass index; CCI, Charlson Comorbidity Index; ISS, Injury Severity Score.

### Dynamics of BMI association with mortality spanning from subacute to chronic SCI

Next, we applied an additional non-linear model (generalized additive model [GAM]) to investigate the association between BMI at admission and long-term mortality. In order to explore if there was a shift over time, we assessed the association between linear BMI and mortality at various time points after SCI in an unadjusted and adjusted GAM. For the outcome of mortality, a restricted cubic spline curve analysis demonstrated a non-linear association of BMI with mortality. This association was visible throughout the time points in the adjusted models (Supplementary Figure S1), whereas, in the unadjusted models, a similar pattern was also detected at 1 and 3 months after SCI. For BMI values less than 17.5 kg/m^2^, the slope was inclined, indicating a higher mortality (Supplementary Figure S1). With a higher BMI >30.5 kg/m^2^, a declined slope at all time points indicates a progressively reduced risk (Supplementary Figure S1).

Further spline curve analysis revealed a non-linear association between mortality and age, where there was increased mortality with higher age and an inclined slope indicating a progressively increased risk (Supplementary Figure S1J).

## Discussion

Mortality risk during acute and chronic phases following SCI (ranging from 1 month to 94 months or 7.7 years) was associated with BMI at admission. Data-driven CART analysis was applied to determine which BMI categories were associated with mortality. Multivariable Cox regression models adjusting for effects of confounders such as age, sex, CCI, ISS, AIS, and neurological level were applied. Finally, spline curve analyses were calculated, depicting the association of BMI at admission with mortality over time after SCI.

The results based on the CART analysis indicated two different effects. There was an overarching strong effect of being underweight (BMI < 17.5 kg/m^2^), which was positively associated with mortality (HR 5.5), and a milder effect of an inverse association of being overweight (BMI > 30.5 kg/m^2^) with mortality (HR 0.28). While being underweight (BMI <17.5 kg/m^2^) was associated with an increased mortality risk, a higher BMI (>30.5 kg/m^2^) may be considered protective. This study suggests a putative ‘obesity paradox’ pronounced during the first months after SCI and diminishing thereafter. Deciphering the mechanisms underlying these protective effects may provide new leads for improving the survival of normal and underweight SCI patients.

Adjusted Cox regression and spline curve analysis confirmed the robustness of the survival analysis. Additionally, the comparison of baseline characteristics among the BMI groups defined by CART did not provide evidence of obvious differences in the composition of the BMI groups that might otherwise explain the differential mortality risk. For example, despite having a slightly higher ISS, which is a predictor of mortality during acute care, the low-range BMI group comprised fewer cases of complete (AIS A) and cervical SCI compared with the mid-range group, both of which are associated with long-term mortality (1). Together, the observed BMI effects were observed independently of either applying predefined BMI or CART categories and emphasized their relevance.

Other recent evidence analyzing multi-center data confirmed that mortality risk is altered in individuals with deviations from “normal” weight, both for patients being overweight and underweight (19). However, the studies have a fundamentally different design and thus are not directly comparable. While Wen et al. focused on a time window ranging from 3 months to 1 year after SCI, this analysis also included an early time window (before 3 months) as well as a long-term endpoint (up to 7.7 years). In addition, Wen et al. measured body weight and height during initial rehabilitation in patients up to 90 days post-injury, and our study used pre-injury or admission body weight and height. Thus, post-injury changes in body weight in the Wen et al. study could explain these divergent results. Furthermore, the analytical strategy was considerably different. Our study did not rely on the WHO criteria developed for able-bodied individuals as it may not be appropriate for individuals with SCI (23), but instead, we used a data-driven, unsupervised approach to identify BMI ranges associated with mortality risk. Notably, the BMI effects were stronger for the recursive partitioning-based categories compared with those based on the WHO definitions, both for underweight (HR 5.5 vs. 2.4) as well as for obese (HR 0.28 vs. 0.32) BMI categories. Moreover, the effects observed in the CART BMI categories were confirmed by the cubic splines within the GAMs. In the unadjusted models, a significant association developed after 1 year to the final data point (7.7 years). In the adjusted models, the association between being underweight and having a greater risk for mortality as well as the protective effects of obesity were visible early on, from 1 month throughout the follow-up period of 7.7. years. Together, these results suggest that being underweight at admission is an extra risk factor compared to what would be expected by a later reduction of BMI only.

Aligned with the subtle protective effect of a higher BMI, its detection may be more difficult and dependent on the array of biostatistical methods being applied. This is supported by ongoing debates in other acute central nervous system (CNS) injury areas such as stroke (28) while more recent high-quality multi-center studies identified an obesity paradox (8). After traumatic brain injury, an obesity-associated decrease in overall complications was observed; however, this did not result in reduced mortality (29). In chronic neurodegenerative disease, a high BMI demonstrated a protective effect regarding disease prevalence (12) and mortality (13). Patients who are malnourished have been considered at higher risk given their lowered metabolic reserve necessary to survive the complications they encounter after injuries such as SCI. For example, even mounting a fever to combat infection poses profound metabolic needs. Future studies using novel techniques are needed to link mortality with better measures of energy expenditure (30). In addition to energy expenditure in underweight individuals, skewed neuroendocrine profiles can trigger muscle wasting and sarcopenia after acute CNS injury (31) pointing to a multifaceted and so far poorly understood area regarding which elements are contributing to systemic pathophysiology that emerges following SCI.

In considering these results, it is important to acknowledge the limitations. This study is not population-based, and as a result, there is a potential bias with regard to the catchment area and the representation of different ethnic minorities or rural populations. We acknowledge that the missing data and use of self-reported data (e.g., height and weight) inherent to using registries may also introduce bias. Nevertheless, relevant differences between included and excluded patients were only observed for AIS. As the included patients comprised more AIS A patients, we do not expect a bias toward an underrepresentation of seriously injured patients in the analysis. Moreover, assessing BMI at admission only is a possible limitation, as BMI is likely to change over time (32). BMI may underestimate the amount of body fat, especially in populations experiencing changes in their body composition, and future studies should explore changes in fat, lean tissue, and bone mineral content. It is also important to acknowledge the small sample size (*n* = 12) for the BMI < 17.5 kg/m^2^ group, and the reported effect estimates should be interpreted cautiously. Future studies are needed with larger samples to validate our results.

While the limitations are inherent, studies investigating acute or neurodegenerative diseases had similar limitations, and the presented data represent the ‘best evidence available’ to substantiate the need for prospective multi-center studies to validate these findings. Systematic studies on changes in body composition after SCI and on treatment options are warranted to establish the pathophysiology and evidence-driven management of nutritional status in these patients, particularly to determine what specific nutritional support might mitigate the risk of mortality in those who are ‘underweight’ when injured. While our article primarily addresses survival during the acute phase and the potentially protective effects of a high BMI, it is important to acknowledge the challenges with weight management and the serious health impacts of chronic SCI.

In conclusion, high BMI imposes a mild protective factor associated with lower mortality in individuals sustaining SCI, concordant with a modest “obesity paradox.” Moreover, being underweight is a highly significant risk factor for death during acute care and up to 7.7 years after SCI. The results suggest unknown neuro-metabolic pathways crucial for survival that are impaired in patients who are underweight. Identifying protective mechanisms and factors underlying the protective effectiveness of adiposity may lead to increased survival in low- to normal-weight patients early after SCI.

## Data Availability

The data analyzed in this study is subject to the following licenses/restrictions: access to deidentified data used for this study is available via the RHSCIR Data Use and Disclosure Policy which is administered by the Praxis Spinal Cord Institute. Requests to access these datasets should be directed to dataservices@praxisinstitute.org.

## Abbreviations

AIS: American Spinal Injury Association Impairment Scale
ASIA: American Spinal Injury Association
BMI: Body mass index
CART: Classification and regression tree
CCI: Charlson Comorbidity Index
CNS: Central nervous system
GAM: Generalized additive model
ISNCSCI: International Standards for the Neurological Classification of Spinal Cord Injury
ISS: Injury Severity Scale
ML: Machine learning
RHSCIR: Rick Hansen Spinal Cord Injury Registry
SCI: Spinal cord injury
WHO: World Health Organization
