# Correction: Effect of body mass index on survival after spinal cord injury

**DOI:** 10.3389/fneur.2025.1667648

**Published:** 2025-11-10

**Authors:** Nader Fallah, Vanessa K. Noonan, Nancy P. Thorogood, Brian K. Kwon, Marcel A. Kopp, Jan M. Schwab

**Affiliations:** 1 Praxis Spinal Cord Institute, Blusson Spinal Cord Centre, Vancouver, BC, Canada; 2 Department of Medicine, University of British Columbia, Vancouver, BC, Canada; 3 Department of Orthopaedics, Vancouver Spine Surgery Institute, University of British Columbia, Vancouver, BC, Canada; 4 International Collaboration on Repair Discoveries (ICORD), University of British Columbia, Vancouver, BC, Canada; 5 Department of Neurology and Experimental Neurology, Clinical and Experimental Spinal Cord Injury Research, Charité – Universitätsmedizin Berlin, Berlin, Germany; 6 QUEST-Center for Transforming Biomedical Research, Berlin Institute of Health, Berlin, Germany; 7 Department of Neurology, Spinal Cord Injury Division, The Ohio State University, Wexner Medical Center, Columbus, OH, United States; 8 Belford Center for Spinal Cord Injury, Departments of Physical Medicine and Rehabilitation and Neuroscience, The Ohio State University, Wexner Medical Center, Columbus, OH, United States

**Keywords:** acute spinal cord injury, body mass index, mortality risk, Charlson comorbidity index, injury severity score

The caption of Figure 2B appears below.

“Linearized cumulative survival over time illustrated a protective effect of a higher BMI in a class (dose) dependent manner which occurs early and is long-lasting. Whereas, elevated mortality was observed in patients who were severely underweight (< 17.5 kg/m^2^, red, *n* = 12), patients with a BMI of 17.5–30.5 kg/m^2^ (green, *n* = 578) or >30.5 kg/m^2^ (blue, *n* = 53) were protected mirrored by a less negative slope that nearly plateaus after 3 years.”

There was a mistake in Figure 2B as published. The colors in the figure did not match the corresponding BMI categories in the legend. The corrected Figure 2B, appears below.

**Figure 2B:**
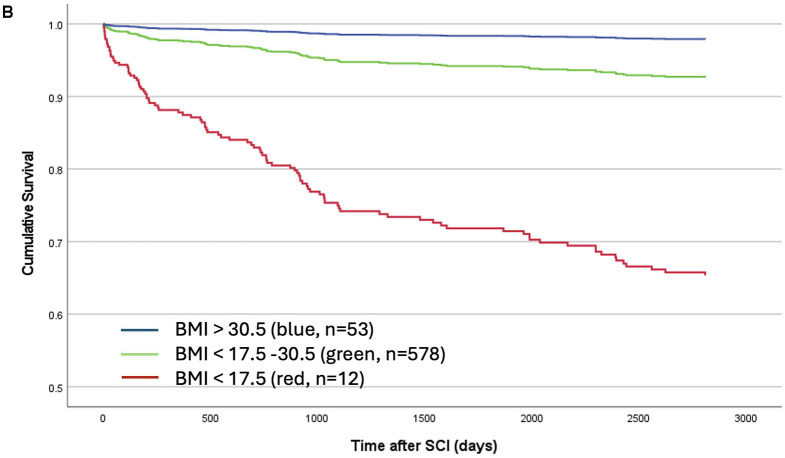


There was a mistake in Supplementary Figure 2 as published. The colors of the lines on the graph did not match the corresponding BMI categories in the legend and caption.

The corrected Supplementary Figure 2, appears below.

**Supplementary Figure 2:**
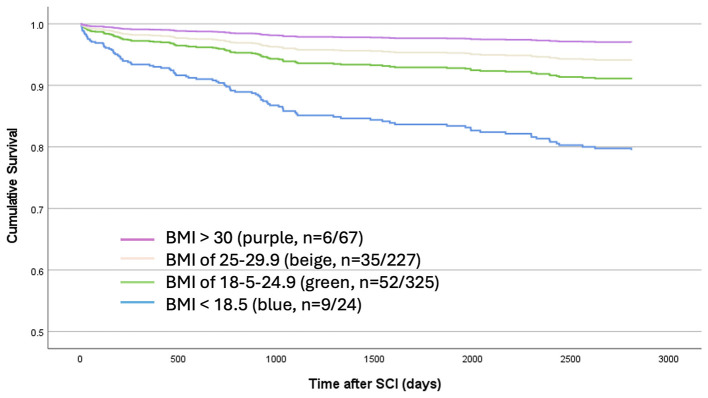


The original version of this article has been updated.

