# Supplementary material for: Effect of body mass index on survival after spinal cord injury
Source: Front Neurol. 2024 Jan 26;14:1269030. doi: 10.3389/fneur.2023.1269030 (PMC10853461; doi:10.3389/fneur.2023.1269030)
Supplement: Supplementary file 1 [file Data_Sheet_1.docx]

***Supplementary Material***

**Effect of body mass index on survival after spinal cord injury**

**Nader Fallah^1,2^, Vanessa K. Noonan^1^, Nancy P. Thorogood^1^, Brian K. Kwon^3,4^, Marcel A. Kopp^5,6^ and Jan M. Schwab^5,7,8*^**

^1^Praxis Spinal Cord Institute, Blusson Spinal Cord Centre, Vancouver, British Columbia, Canada

^2^Division of Neurology, Department of Medicine, University of British Columbia, Vancouver, British Columbia, Canada

^3^Vancouver Spine Surgery Institute, Department of Orthopaedics, University of British Columbia, Vancouver, British Columbia, Canada

^4^ICORD (International Collaboration on Repair Discoveries), University of British Columbia, Vancouver, British Columbia, Canada

^5^Department of Neurology and Experimental Neurology, Clinical and Experimental Spinal Cord Injury Research, Charité - Universitätsmedizin Berlin, Germany

^6^Berlin Institute of Health (QUEST-Center for Transforming Biomedical Research), Berlin, Germany

^7^Department of Neurology, Spinal Cord Injury Section, The Ohio State University, Wexner Medical Center, Columbus, USA

^8^Belford Center for Spinal Cord Injury, Departments of Physical Med. & Rehabilitation and Neuroscience, The Ohio State University, Wexner Medical Center, Columbus, Ohio, USA

*** Correspondence:** Jan M. Schwab: [Jan.Schwab@osumc.edu](mailto:Jan.Schwab@osumc.edu)

1. **Supplementary Figures and Tables**
   1. **Supplementary Figures**

**Supplementary Figure 1.** Adjusted and unadjusted relationships between mortality and age or BMI

(relative Hazard) at various time points from acute to chronic SCI. (A-J). The relationship between

BMI as continuous variable at admission and mortality was assessed using unadjusted and adjusted

analyses at (**(A),(B))** one month, **((C),(D))** 3 months, **((E),(F))** 1 year and **((G),(H))** 7.7 years (long-term) after SCI. While there was no significant relationship between BMI and one-month mortality or three-month mortality, it was significant at one-year (p<0.004) and 7.7 years (p<0.05); overall there was an association between BMI and mortality. Using a spline function to investigate the shape of the association, it revealed a concave shape at all timepoints, where the association strengthened with time after SCI. Overall, while the curve with a BMI of approximately 17.5 kg/m^2^ increases (positive slope) the risk of mortality while a BMI > 30.5 kg/m^2^ is associated with a lower mortality (negative slope). Body mass index was calculated as kg/m^2^. (I and J) Relationship between age and risk of mortality for the full 7.7 year follow up. The mortality risk increased with age (Hazard risk ratio, x-axis: BMI at admission).


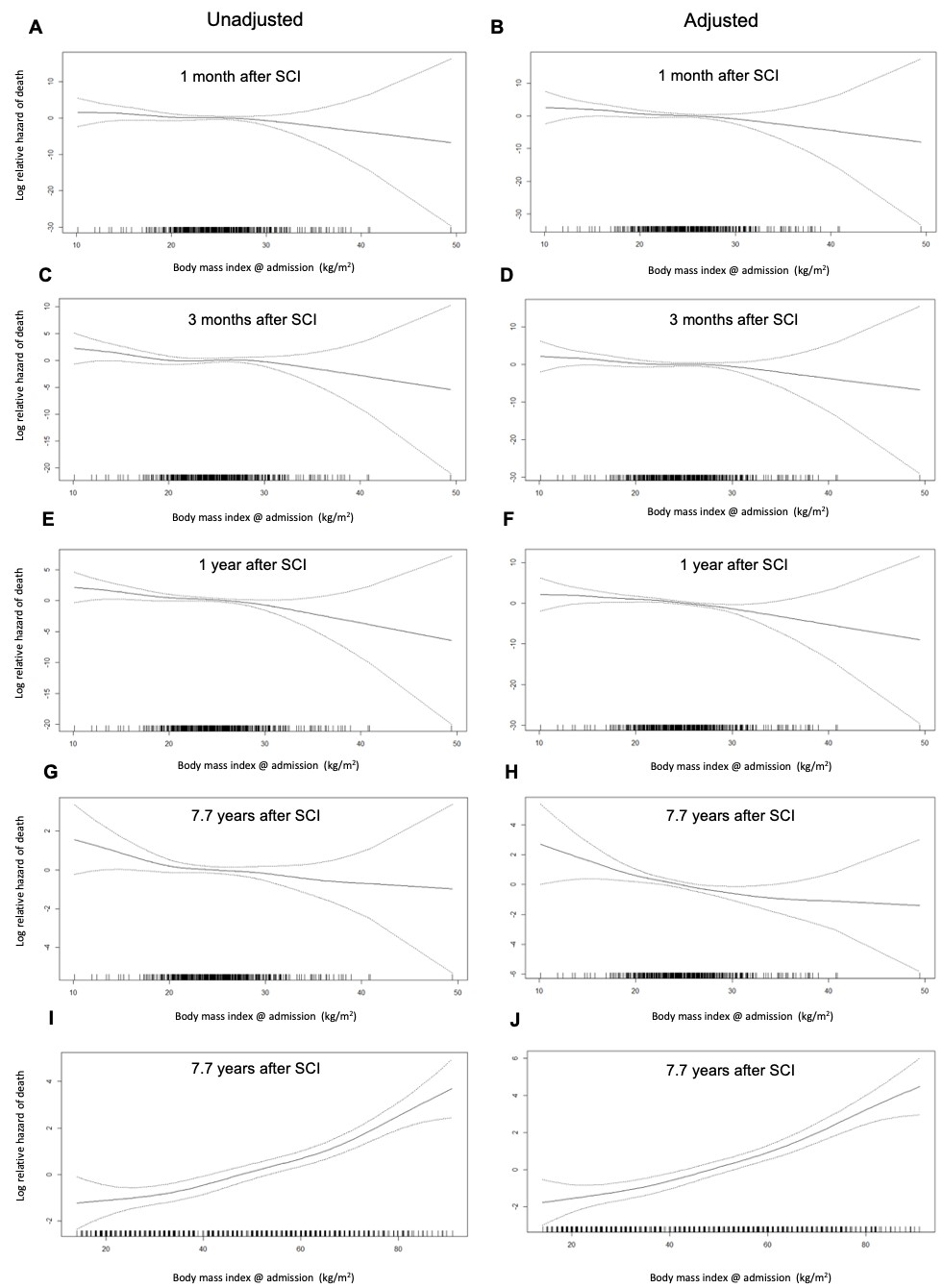


**Supplementary Figure 2**. Cumulative survival analysis stratified by WHO BMI categories. Cumulative survival over time confirmed a protective effect of BMI in a class (dose) dependent manner to occur early and being long lasting. Mortality risk rapidly progressed in patients who were underweight (<18.5 kg/m^2^, blue, n=9/24) compared to patients with a BMI in the normal/medium range (18.5 – 24.9 kg/m^2^, green, n=52/325); however, patients who were overweight (25.0 – 29.9 kg/m^2^, beige, n=35/227) or obese > 30 kg/m^2^ (purple, n=6/67) had a lower mortality risk (demonstrated a less steep decline).


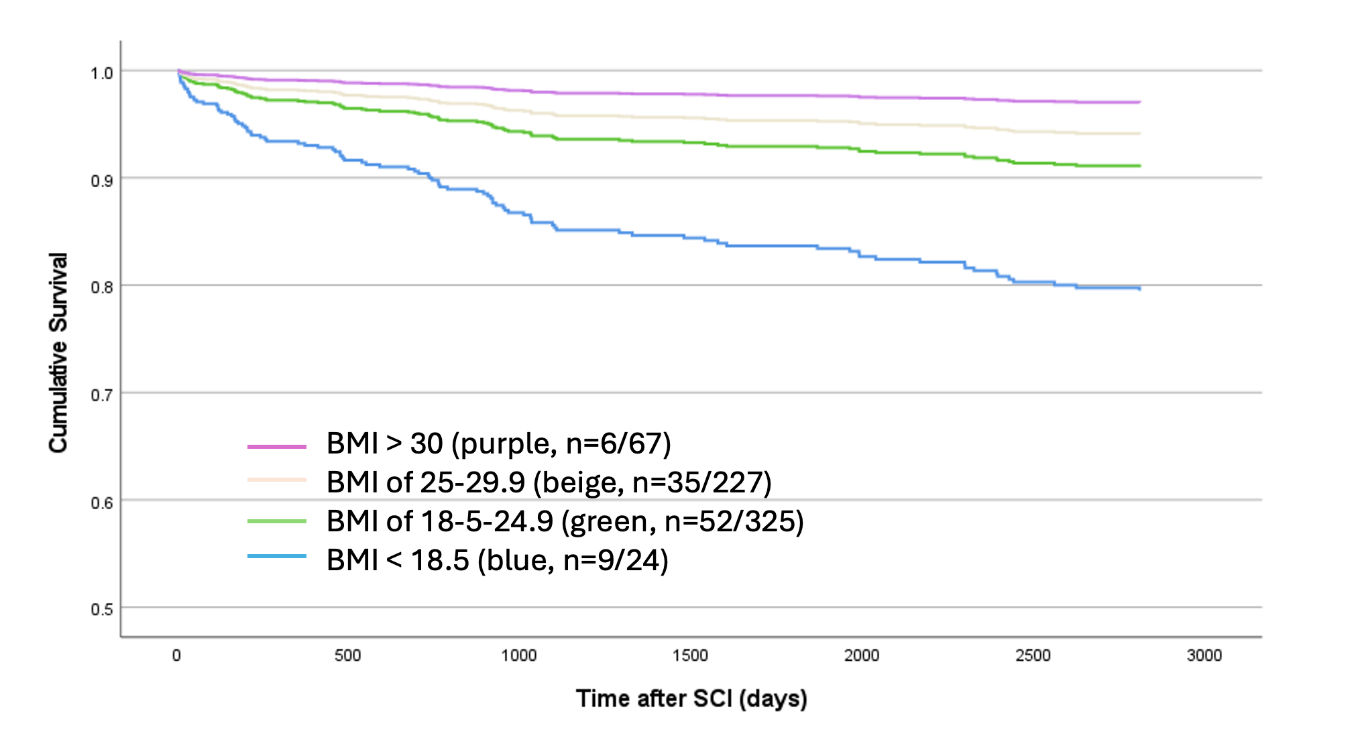


- 1. **Supplementary Tables**

**Supplementary Table 1.** Mortality risk associated with body mass composition (BMI) according to the WHO obesity criteria and categories. The underweight and obese groups were significantly different compared to the normal BMI group. Compared to the normal BMI group, the mortality risk was significantly (p = 0.01) reduced to 32% in the obese group (BMI > 30.0 kg/m^2^). By contrast, individuals who were underweight (BMI < 18.5 kg/m^2^) had a significantly (p=0.017) elevated, 2.4-fold mortality risk when compared to the normal BMI group (BMI 18.5 -24.9 kg/m^2^).

| **Model** | **Variables** | **Hazard Ratio** | **95% CI** | **p-value** |
| --- | --- | --- | --- | --- |
| **Univariable** | BMI Normal 18.5-24.9 kg/m^2^ (ref) |  |  |  |
|  | BMI: Malnutrition (<18.5 kg/m^2^) | 2.71 | 1.83-9.57 | < 0.001 |
|  | BMI: Overweight (25.0-29.9 kg/m^2^) | 0.96 | 0.63-1.47 | 0.85 |
|  | BMI: Obese (>30.0 kg/m^2^) | 0.53 | 0.23-1.23 | 0.53 |
| **Multivariable** | Age (per 1-year increase) | 1.07 | 1.06 – 1.08 | < 0.001 |
|  | Sex (male) | 3.22 | 1.87 – 5.54 | < 0.001 |
|  | Neurological level (cervical) | 4.29 | 2.25 – 8.19 | < 0.001 |
|  | AIS A | 3.10 | 1.64 – 5.82 | < 0.001 |
|  | AIS B | 2.25 | 1.08 – 4.65 | 0.029 |
|  | AIS C | 1.60 | 0.80 – 3.19 | 0.185 |
|  | ISS (per 1unit increase) | 1.02 | 1.01 –1.03 | 0.002 |
|  | BMI Normal 18.5-24.9 kg/m^2^ (ref) |  |  |  |
|  | BMI: Malnutrition (<18.5 kg/m^2^) | 2.43 | 1.17-5.03 | 0.017 |
|  | BMI: Overweight (25.0-29.9 kg/m^2^) | 0.65 | 0.42-1.01 | 0.053 |
|  | BMI: Obese (>30.0 kg/m^2^) | 0.32 | 0.14-0.76 | 0.009 |

AIS, American Spinal Injury Association Impairment Scale; ISS, Injury Severity Score; BMI, Body Mass Index; WHO, World Health Organization.

WHO BMI Categories: BMI <18.5 kg/m^2^ (N = 24); BMI 18.5-24.9 kg/m^2^ (N = 325); BMI 25.0-29.9 kg/m^2^ (N=227); BMI >30.0 kg/m^2^ (N = 67).

**Supplementary Table 2**. Mortality risk associated with an increase in BMI as a continuous variable. A sensitivity analysis using BMI as a continuous variable demonstrated a protective effect (reduced mortality risk). A one unit increase in BMI is associated with a 9% reduction in mortality risk.

| **Variables** | **Hazard Ratio** | **95% CI** | **p-value** |
| --- | --- | --- | --- |
| Age (per 1-year increase) | 1.07 | 1.06 – 1.08 | < 0.001 |
| Sex (male) | 3.4 | 1.97 – 5.87 | < 0.001 |
| Neurological level (cervical) | 4.23 | 2.23 – 8.05 | < 0.001 |
| AIS A | 3.12 | 1.66 – 5.86 | < 0.001 |
| AIS B | 2.24 | 1.08 – 4.64 | 0.029 |
| AIS C | 1.63 | 0.82 – 3.24 | 0.167 |
| ISS (per 1-unit increase) | 1.02 | 1.01 –1.03 | 0.001 |
| BMI kg/m^2^ | 0.91 | 0.86 – 0.96 | < 0.001 |

AIS, American Spinal Injury Association Impairment Scale; BMI, Body Mass Index; ISS, Injury Severity Score

**Supplementary Table 3**. Association between BMI category, neurological level and morality. In summary, within the stratum of patients with neurological level C1-T1, the effects observed in the overall sample are confirmed. We observe a higher mortality in the underweight group and a slightly lower mortality in the obese group. In the T2-S5 stratum, the effects are difficult to interpret because of the lower mortality rate and thus lower number of events. However, since we observe a similar pattern at least with regards to underweight, it appears reasonable to evaluate the total sample. The notion of a lesion-level dependent effect on mortality is important. Further light on the underlying mechanism is provided by recent studies. In brief, lesion-level dependent hypercortisolism (increased after high compared to low level SCI) has been a causative driver of muscle wasting, affecting muscle above and below the lesion side after acute/subacute SCI (1). Hence, as muscle wasting/sarcopenia is contributing considerably to overall weight loss, the lesion-level dependent neuroendocrine tone is a lesion level dependent mechanism resulting into catabolism and therefore a candidate driver for enhanced mortality risk. Lesion level dependent hypercortolism has been associate with autonomic instability and a sympathetically ‘decentralized’ adrenal gland (2).

| **C1-T1** | | | | |
| --- | --- | --- | --- | --- |
|  |  | **Survive** | **Mortality** | **Total** |
| Normal | Count | 158 | 48 | 206 |
|  | Percent | 76.7% | 23.3% | 100.0% |
| Malnutrition | Count | 8 | 7 | 15 |
|  | Percent | 53.3% | 46.7% | 100.0% |
| Overweight | Count | 116 | 32 | 148 |
|  | Percent | 78.4% | 21.6% | 100.0% |
| Obese | Count | 36 | 4 | 40 |
|  | Percent | 90.0% | 10.0% | 100.0% |
| **T2-S5** | | | | |
| Normal | Count | 115 | 4 | 119 |
|  | Percent | 96.6% | 3.4% | 100.0% |
| Malnutrition | Count | 7 | 2 | 9 |
|  | Percent | 77.8% | 22.2% | 100.0% |
| Overweight | Count | 76 | 3 | 79 |
|  | Percent | 96.2% | 3.8% | 100.0% |
| Obese | Count | 25 | 2 | 27 |
|  | Percent | 92.6% | 7.4% | 100.0% |

**References**

1. Harrigan ME, Filous AR, Vadala CP, Sahenk Z, Prüss H, Reiser PJ, et al. Spinal cord injury induces early systemic muscle wasting mediated by lesion-level dependent neuroendocrine tone. Sci Transl Med. 2023;In press.

2. Prüss H, Tedeschi A, Thiriot A, Lynch L, Loughhead SM, Stutte S, et al. Spinal cord injury-induced immunodeficiency is mediated by a sympathetic-neuroendocrine adrenal reflex. Nat Neurosci. 2017;20:1549–59.
